# Mechanism of Human Telomerase Reverse Transcriptase (*hTERT*) Regulation and Clinical Impacts in Leukemia

**DOI:** 10.3390/genes12081188

**Published:** 2021-07-30

**Authors:** Mot Yee Yik, Adam Azlan, Yaashini Rajasegaran, Aliaa Rosli, Narazah Mohd Yusoff, Emmanuel Jairaj Moses

**Affiliations:** Regenerative Medicine Sciences Cluster, Advanced Medical and Dental Institute, Universiti Sains Malaysia, Kepala Batas 13200, Malaysia; yymot81@gmail.com (M.Y.Y.); muhdadamazlan@yahoo.com (A.A.); yaashini@student.usm.my (Y.R.); aliaaarosli@gmail.com (A.R.); narazah@usm.my (N.M.Y.)

**Keywords:** *hTERT*, leukemia, gene regulation, cancer, hematological malignancy

## Abstract

The proliferative capacity and continuous survival of cells are highly dependent on telomerase expression and the maintenance of telomere length. For this reason, elevated expression of telomerase has been identified in virtually all cancers, including leukemias; however, it should be noted that expression of telomerase is sometimes observed later in malignant development. This time point of activation is highly dependent on the type of leukemia and its causative factors. Many recent studies in this field have contributed to the elucidation of the mechanisms by which the various forms of leukemias increase telomerase activity. These include the dysregulation of telomerase reverse transcriptase (*TERT*) at various levels which include transcriptional, post-transcriptional, and post-translational stages. The pathways and biological molecules involved in these processes are also being deciphered with the advent of enabling technologies such as next-generation sequencing (NGS), ribonucleic acid sequencing (RNA-Seq), liquid chromatography-mass spectrometry (LCMS/MS), and many others. It has also been established that TERT possess diagnostic value as most adult cells do not express high levels of telomerase. Indeed, studies have shown that prognosis is not favorable in patients who have leukemias expressing high levels of telomerase. Recent research has indicated that targeting of this gene is able to control the survival of malignant cells and therefore offers a potential treatment for TERT-dependent leukemias. Here we review the mechanisms of *hTERT* regulation and deliberate their association in malignant states of leukemic cells. Further, we also cover the clinical implications of this gene including its use in diagnostic, prognostic, and therapeutic discoveries.

## 1. Introduction

The human telomerase reverse transcriptase gene (*hTERT*) spans over a 40 kb DNA region and consists of 16 exons and 15 introns [1]. This gene produces a polypeptide with a length of 1132 amino acids which is then transformed to a 130kD functional TERT protein [2]. The four critical functional domains in TERT comprise the N-terminal regulatory domain, the RNA binding domain, the reverse transcriptase domain, and the C-terminal dimerization domain [3]. Owing to its complexity, *TERT* is regulated at various levels which include transcriptional, post-transcriptional, and post-translational mechanisms [4,5,6].

The primary function of this protein is in the maintenance of telomere length. Telomeres cap the ends of chromosomes with repetitive 5′-TTAGGG-3′ conserved sequences to maintain the integrity of genetic information [7]. With each successive round of cell proliferation, 50 to 200 base pairs of DNA material are dissociated from the ends of the chromosomes due to the loss of RNA primers situated on the lagging strand of Okazaki fragments. This phenomenon leads to the progressive shortening of telomeres which limits the potential of somatic cells to divide [8,9]. Certain cell types, such as stem cells and cancer cells, are able to express telomerase to replenish telomeric ends, thereby permitting continuous self-renewal [10,11,12]. Telomerase exerts its DNA polymerizing function through the formation of a complex consisting of two main subunits: the catalytic protein hTERT and the human telomerase RNA component (hTERC) subunit [13].

*TERT* dysregulation has been identified as a common aberration in leukemogenesis [14]. All major forms of leukemia including acute myeloid (AML), acute lymphoid (ALL), chronic myeloid (CML), and chronic lymphoid (CLL) leukemia have implicated TERT as an essential factor for the development and progression of the disease [15,16,17,18]. The mediators involved in the dysregulation of *TERT* have been extensively studied and the mapping of their contribution to pathological pathways have been reported in the various forms of leukemia as well [19].

This article reviews the latest information in the field of *TERT* regulation and its influences on leukemogenesis. This would include detailed information on the transcriptional and translational regulation of *TERT*, the dysregulation of *TERT* in leukemias, and the clinical implications of this gene in the treatment of these disorders.

## 2. Transcriptional Regulation of *TERT*

The promoter of *TERT* is a very dynamic region. It is GC rich and lacks the TATA or CCAAT recognition sequence for the transcription start site (TSS) location by RNA polymerase 11. However, this is compensated for by an initiator like CCTCTCC sequence. It also contains two E boxes for Myc binding, several GC boxes for Sp1 binding, and a CCAC box [6]. These motifs make the promoter region an excellent hub for the binding of various repressors and activators [5,6].

*TERT* expression in adult humans are limited to stem cell, germline, and tissues with high turnover rates. It has been disclosed that the highest levels are found in testicular tissues which give rise to spermatocytes [10,20], intestinal crypts where shedding of cells are excessive [21], and hepatocytes which repopulate the liver during homeostatic conditions as well as in mitigation of injury [12]. *TERT* expression is not normally observed in adult somatic cells and their atypical occurrence is usually due to *TERT* promoter mutations leading to the abrogation of telomerase silencing [22]. This phenomenon is highly associated to the development of various forms of cancers including leukemia.

### 2.1. Negative Transcriptional Regulators of TERT

Negative transcriptional regulators of *TERT* can either bind directly to the promoter or interact by forming complexes to downregulate *TERT* expression. Direct acting repressors include retinoblastoma (RB), myeloid-specific zinc finger protein (MZF2), activator protein 1 (AP1), Wilms tumor 1 (WT1), and menin [23,24,25]. Transcriptional repressors exert their effect on *TERT* expression by forming complexes and interacting with other proteins including Mad1, the HTLV-1 oncogene TAX, tumor suppressors BRCA1/Nmi and p53, interferon regulatory factor 1 (IRF1), and transforming growth factor β (TGFβ) [26,27,28,29,30]. Mad1 has been identified to form a complex with Max (Mad1/Max) and binds to the E box region located at the promoter to prevent *TERT* expression [26,31,32]. Similarly, it was found that TAX also represses *TERT* expression by E-box binding [27]. Receptor Ck, on the other hand, has been determined to regulate *TERT* via interactions with protein kinase C [33]. In other studies, BRCA1/Nmi was found to interfere with c-Myc binding while p53 disrupts Sp1 binding [28,34,35]. IRF1 suppresses *TERT* expression via tumor suppressor CDKN1B (p27) [29], while TGFβ was found to interact with SMad3 and SIP1 to downregulate *TERT* [30,36,37].

### 2.2. Positive Transcriptional Regulators of TERT

Positive transcriptional regulators also function via direct and indirect interactions with the promoter region to activate *TERT* expression. Positive regulators that act directly include human N-acetyltransferase like protein (hALP), transcriptional elements interacting factor (TEIF), signal transducer and activation of transcription 3 (STAT3), the oncoproteins Ews-ETS, hypoxia inducible factor 1 (HIF1α), and the transcription factor c-Jun [5]. Activators that act indirectly include the c-Myc oncogene, the apoptosis inhibitor survivin, and the viral oncogene E6/NFX1. The oncogene c-Myc activates *TERT* expression by forming a complex with max (c-Myc/Max) and binding to the E-box region whilst survivin acts via SP1/c-Myc binding. The viral oncogene E6/NFX1 on the other hand induces *TERT* expression by p53 degradation, activation of c-Myc, and disruption of upstream stimulatory factor (USF) based repression [38,39,40].

### 2.3. Conditional Transcriptional Regulators of TERT

There are some regulators, such as transcription factors E2F and specificity protein 1 (Sp1), upstream stimulatory factors (USF1/2), and tumor suppressor p73, which act as both repressors and activators of *TERT* expression depending on the condition. E2F acts as a *TERT* repressor in cancer cells; however, the condition is reversed in normal cells where it activates the expression of *TERT*. This transcription factor controls *TERT* regulation by binding to the GC box region on the promoter [41,42]. USF1/2 on the other hand acts as a repressor in normal cells and activator in cancer cells. This is achieved by E box binding on the promoter [43,44]. Sp1 acts as a repressor of *TERT* in cancer cells by forming a complex with Sp3, while in Kaposi sarcoma associated herpes virus (KSHV), Sp1 becomes an activator via interactions with latency-associated antigen—briefly known as LANA [45,46,47]. The tumor suppressor p73 also interacts with Sp1 to suppress *TERT* while activation of *TERT* is mediated by p73β expression [48,49,50].

## 3. Post Transcriptional Regulation of *TERT*

*hTERT* has been widely studied for the past few decades and the discoveries relating to its mechanisms of action have established the importance of *hTERT* in cancer biology. In line with these observations, the regulation of *hTERT* at post-transcriptional level has demonstrated close association with mRNA alternative splicing and non-coding RNA functions [13,51,52,53,54]. However, the regulatory mechanisms and functions of alternative splicing in *hTERT* are not well understood [53]. Regulation of *hTERT* by microRNA (miRNA) has recently contributed to the understanding of the diverse regulatory mechanisms of *hTERT* [53,54,55]. MicroRNAs play a key role in post-transcriptional regulation as gene regulators of their target gene [13,51,53]. Based on previous reports on miRNAs targeting *hTERT*, miRNAs are able to act as tumor suppressors or oncogenes depending on their target gene [13,51,52]. Interestingly, *hTERT* regulation in different cancers has been shown to be regulated by different miRNAs. Collectively, it has been established that every miRNA involved with *hTERT* regulation works synchronously with different regulators of the target genes.

### TERT Regulation by microRNAs

MicroRNAs (miRNAs) are short endogenous non-coding RNA molecules consisting of 20–24 nucleotides loaded onto Argonaute (Ago) proteins to regulate gene expression [51,56]. They are transcribed by RNA polymerase II and processed by nuclear and cytoplasmic enzymatic complexes to yield their mature forms. The matured miRNA strand that remains incorporated into the Argonaute-containing RNA-induced silencing complex (RISC) modulates the post-transcriptional gene silencing of target genes [57,58,59]. These complexes provide imperfectly complementary sites for base-pair interactions with the targeted mRNA molecules inducing translational repression or mRNA cleavage [13,60]. The mechanisms associated with miRNA biogenesis and its influence on the *hTERT* gene have made substantial progress as evidenced by recently published studies. It has been reported that miRNAs can post-transcriptionally alter *hTERT* transcripts directly or indirectly [13,54]. MiRNAs can directly bind to conserved *hTERT* complementary sites located in the 3′-untranslated region (3′UTR) to restrict *hTERT* expression by negatively regulating their translation [61,62,63]. Specifically, expression of specific miRNAs have been shown to exert changes in tumor cell proliferation, cell cycle control, apoptosis, metastatic invasion, and protein expression levels [53,59]. Each miRNA has the potential to mediate the expression of their target genes. miR-1182 and miR-491-5p are two miRNAs that directly target the transcript of *hTERT* [64,65]. These interactions have been confirmed via luciferase assay. Further, it has been elucidated that miR-491-5p is involved in the regulation of the *PI3K*/*AKT* signaling pathway. Therefore, together these epigenetic gene regulators influence hTERT gene expression in cancers. In contrast, miRNAs may also indirectly target the transcription factors involved in *hTERT* regulation [13]; however, the precise molecular mechanisms that underlie post-transcriptional repression are still unclear.

## 4. Post Translational Regulation of *TERT*

Post-translational modifications of hTERT could modify protein stability, subcellular localization, and ultimately, enzyme activity [53]. To date, studies have identified that post-translational regulation of *hTERT* mainly involve phosphorylation and ubiquitination by kinases and ligases respectively [4,53,66]. Both phosphorylation and ubiquitination give rise to prominent effects by positively and negatively regulating *hTERT* activity [67].

In phosphorylation, kinases such as protein phosphatase 2A (PP2A) interact with c-Abl tyrosine kinase protein, leading to a threefold reduction of telomerase activity by dephosphorylation [53,68,69]. On the contrary, protein kinase B (an AKT kinase) increases telomerase activity through TERT phosphorylation-dependent activation of the PI3K/Akt/mTOR pathway [68,69]. Similar to protein kinase C, they phosphorylate hTERT by PKC isoenzymes α, β, δ, ε, and ζ to enhance telomerase activity, which is increased along with nuclear accumulation of hTERT [61,66,69,70,71]. In addition, kinases including kinase interacting protein (KIP), c-jun, and mitogen activated protein kinase (MAPK) also regulate post-translational modifications of hTERT. KIP regulation involves interactions with the upstream kinase domain of DNA–PKcs to improve telomerase activity in human cells, while, MAPK upregulate *hTERT* in a serum and pH-dependent manner within the hypoxic environment of solid tumors [5,72].

Among the regulators reported to modify *hTERT* translation through ubiquitination are Makorin RING finger protein 1 (MKRN1), SMAD specific E ubiquitin protein ligase 2 (Smurf2), CHIP (C terminus of Hsc70-interacting protein), E6AP, and the E6 ubiquitin ligase proteins. MKRN1 and Smurf2 are E3 ubiquitin ligases, which play a role as negative regulators of *hTERT*. Overexpression of MKRN1 results in decreased telomerase activity and shortens telomeres through ubiquitination and subsequently dampens *hTERT* expression in vitro and in vivo [53,67,69]. Similar to MKRN1, Smurf2 suppresses telomerase activity through ubiquitination [5,73,74]. C terminus of Hsc70-interacting protein (CHIP), a co-chaperone of E3 ubiquitin ligase, has been identified through its interaction with Hsc70. Through their interaction with hTERT in the cytoplasm, their cellular abundance has also been shown to mediate ubiquitination and degradation, thus suppressing telomerase activity [53,69,75]. In addition, E6AP and the E6 ubiquitin ligase proteins have been shown to function in tandem with hTERT to induce and repress telomerase activity. The interaction between E6AP and NFX-123 mediates ubiquitination which subsequently leads to the reduction of telomerase activity, while E6 ubiquitin ligase proteins reverses repression of the *hTERT* gene by targeting NFX1091 for degradation [5]. In addition, new discoveries have identified sumoylation, methylation, and acetylation as emerging modifications involved in the post-translational regulation of *hTERT* [53]. DNA methylation is a complex topic with recent discoveries proposing different dysregulation of *hTERT* in leukemia especially on the promoter and proximity regions, which is further discussed in Section 5.

More recent studies on non-coding RNAs have identified that this class of regulatory RNAs play vital roles in influencing the dysregulation of *hTERT*. Regulation of *hTERT* via miRNAs are often mediated through indirect mechanisms by targeting upstream proteins. The work of Pourbagheri-Sigaroodi et al. (2019) revealed that *TERT* expression is regulated by miRNAs, which control the inhibition of NF-κB [76]. Additionally, Liu and colleagues (2012) elucidated that hsa-miR-101 regulates the expression of *p*23, a heat shock protein 90 cochaperone essential for telomerase assembly and telomere length maintenance [77]. Another miRNA, miR-196b was also identified which exerts its regulatory effects on *hTERT* via downregulation of c-*myc* [78]. In terms of long non-coding RNAs, H19 was found to regulate hTERT via disrupting the interaction between protein moieties of hTERT and hTR [79].

## 5. TERT Dysregulation in Leukemias

Studies on the various forms of leukemia have disclosed the dysregulation of *TERT* gravely affects the prognosis of the disease and is known to exert its mechanism of action via a plethora of modifications including epigenetics, mutations, amplifications, structural variants, and influences on oncogenes. The prevalence of *TERT* dysregulation remains to be determined in each subtype of leukemia and is also highly dependent on the population and recruitment criteria of each study. An early study has indicated that it affects approximately half of AML (53.3%) patients [80], while another report indicated that *TERT* dysregulation is present in all acute promyelocytic leukemia (APL, AML-M3) patients in their cohort [81]. Other studies have shown that *hTERT* dysregulation was only observed in limited cases of relapsed childhood ALL [82], while a study in Saudi Arabia disclosed that *hTERT* mutations are not linked to predisposition to childhood acute leukemia [83]. In terms of relevance in disease pathogenesis, studies have shown that in adult T-cell leukemia/lymphoma (ATLL), the activation of telomerase is required for the disease development and progression, while in AML and CML it is only required for maintenance and not initiation [15,84,85]. In recent years, there have been many reports highlighting clinical implications and the importance of this enzyme, which includes targeting of TERT by drugs and biological molecules and the interactions of TERT with chemotherapy drugs.

### 5.1. Chronic Myeloid Leukemia (CML)

**Figure 1:**
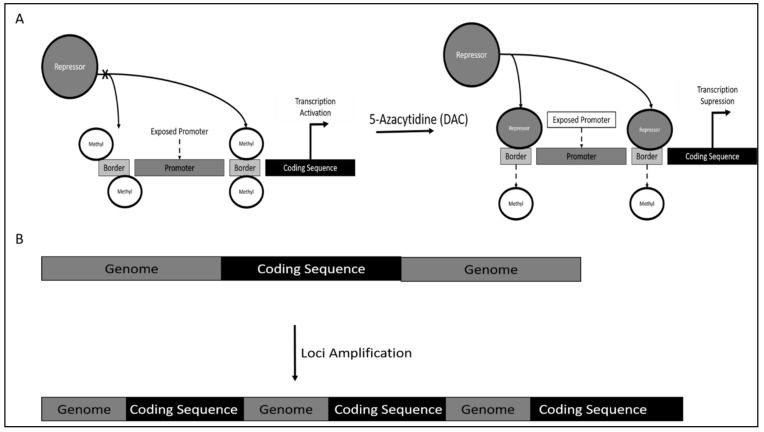
(**A**) Epigenetic regulation of *TERT* via promoter border region methylation. (**B**) Loci amplification of *TERT* gene resulting in an increase in copy number.

Mechanism of *TERT* dysregulation was reported to occur via *TERT* promoter methylation. However, contrary to canonical research, DNA methylation would increase TERT activity, as methylation occurs on the genomic region bordering the *TERT* promoter. This results in the inaccessibility of repressor on this site, which is proximal to the *TERT* promoter, thereby increasing the affinity of *TERT* promoter binding of crucial activators leading to *TERT* hyper-expression (Figure 1A). The observation mentioned above occurred when the CML model cell line K562 was treated with 5-azacytidine (DAC), a known DNA demethylase. Treatment reduced the methylation of *TERT* promoter regions, which are sites for CTCF (Transcription Repressor) binding. The result suggested an increase in CTCF binding, which could be seen from the reduction of C-Myc (Transcription Activator) binding and subsequent downregulation of *TERT* transcription. This further led to TERT reduction induced senescence in chronic myeloid leukemia [86].

Dysregulation of *TERT* also occur via gene amplification, which results in an increase in gene copy number (GCP). This phenomenon translates to increased gene expression levels due to relatively higher abundance of mRNA resulting from amplification (Figure 1B). It was observed that TERT catalytic components were amplified in patients with CML. Interestingly, TERC amplification does not correlate with resistance towards imatinib mesylate, although the copy number was calculated to be above 3 [87].

The CML fusion oncogene *BCR*-*ABL* was also found to contribute to the overall activity of TERT in aiding disease progression. It was reported that the fusion construct controls *hTERT* at different levels, namely at the nuclear entry level, direct TERT activity regulation, and TERT mRNA expression levels. BCR-ABL tyrosine kinase activity inhibition via Gleevec (Tyrosine Kinase Inhibitor) treatment resulted in the accumulation of TERT in the nucleoplasm when compared to the nontreated group in which TERT accumulated within the nucleolus. Furthermore, telomeric length was also found to be implicated following Gleevec treatment of CML cells. It was observed that K562 accumulates short telomeric ends (1 Kb<) when comparing cells treated with Gleevec to non-treated cells. The results were also consistent with hTERT phosphorylation levels where a decrease in phosphorylation states were observed post Gleevec treatment suggesting that hTERT activity is induced by BCR-ABL via direct hTERT phosphorylation. Additionally, transcription of hTERT was also found to be mediated via BCR-ABL tyrosine kinase activity as it was found that Gleevec treatment reduced *hTERT* mRNA abundance in K562. Further experimentation reported that, this was mediated via BCR-ABL activation of STAT5a through phosphorylation, which resulted in STAT5a-Phos binding to the *TERT* promoter. This was further confirmed via STAT5a inhibitor treatment, which significantly reduced *TERT* expression. Luciferase activity assay confirmed that the full length contains STAT5a binding as co-expression of the reporter with constitutive plasmid baring STAT5a resulted in over a twofold increase in *hTERT* expression [88]. Additionally, the same mechanism of STAT activation via BCR-ABL was also observed in CML treated with pimazode, which deactivated STAT5 by inhibiting tyrosine kinase activity of BCR-ABL [89].

**Figure 2:**
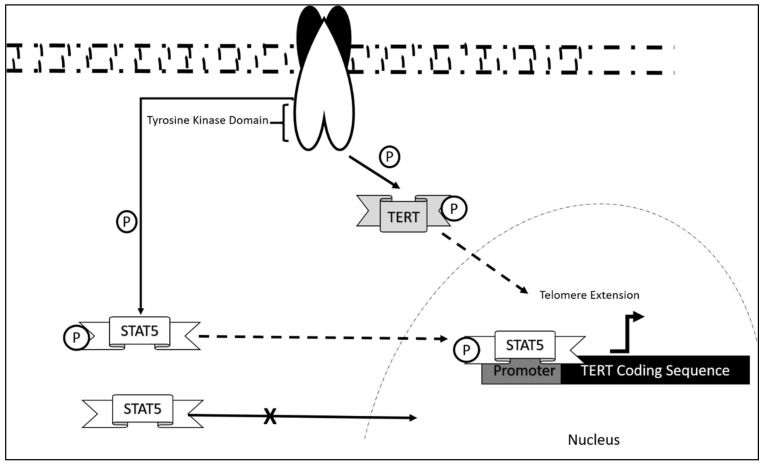
BCR-ABL control of TERT activity and expression generally targeted via Imatinib Mesylate or Tyrosine Kinase Inhibitor treatment.

*TERT* was targeted indirectly through upstream factors crucial for *TERT* activation via inhibition of kinase activity. Imatinib mesylate (IM) is currently one of the major drugs used in CML treatment. However, treatment of CML using IM was found to potentiate CML towards leukemogenesis as it was reported that it enhances expression of *hTERT* via phosphorylation of STAT5 [90] (Figure 2). Furthermore, knockdown of STAT5 attenuated leukemogenesis and increased cells sensitivity towards IM. It was also observed that patients with CML have different levels of phosphorylated STAT, further reducing the efficacy of IM. Further experimentation also reported that this resistance was conferred by the expression of MDR1 activation via STAT5 phosphorylation. Therefore, it is crucial to determine the expression levels of MDR1 and the phosphorylation state of STAT5 for proper treatment of IM in patients with CML [91].

Interestingly, it was also reported that CML cell line K562 with hTERT depletion does not result in telomere shortening post IM treatment. However proliferative potential of CML was significantly attenuated when telomerase depleted cells were treated with IM, further confirming the role of TERT in conferring treatment resistance towards IM [92]. However, treatment of CML with herbamycin A, also a tyrosine kinase inhibitor, was found to be ineffective in *TERT* suppression, which is likely due to the compensatory effects of *TERT* regulators. Nevertheless, herbamycin A treatment of K562 positive TERT cells post cyclin D1 transfection attenuated telomerase activity, while c-myc transfection resulted in partial restoration of hTERT activity [93]. It is therefore crucial to determine the mechanistics of *TERT* activators before IM prescription as differential expression of transcription factors are crucial in CML progression.

Since it is crucial to target *TERT* in senescence induction of CML, indirect regulation via IM would result in the ineffective inactivation of upstream factors as *TERT* is a downstream effector of BCR-ABL, STAT5, and c-MYc. Although the activity of these upstream factors could be effectively modulated by IM, it is of utmost importance to understand that *TERT* is not an exclusive target for these activators. Therefore, CML treatment with IM would be rendered ineffective via other downstream C-Myc or STAT5 target compensation. In this case, *TERT* direct downregulation could be synergistically employed along with IM treatment in order to improve CML treatment. Direct modulation of *TERT* could be employed via the use of BIBR1532, a potent TERT inhibitor which binds directly to TERT core preventing TERT binding onto telomeres. Although lacking studies in CML, it was proven that BIBR1532 is effective in suppressing disease progression in Burkitt’s lymphoma (BL) via direct *TERT* suppression. Co-treatment with fludarabine was also found to potentiate apoptosis in BL treated with BIBR1532 [94]. This synergistic approach of targeting *TERT* with BIBR1532 and IM could serve as a potential treatment for CML.

### 5.2. Chronic Lymphoid Leukemia (CLL)

The mechanism of dysregulation of *TERT* in CLL is still largely unexplored. However, correlation studies report that telomeric length serves as an initial prognostic marker in CLL disease progression [95]. This was also associated with telomerase/telomere associated proteins expression, which also contribute as markers in the diagnosis of CLL onset. Research showed that significant decrease in telomeric length was observed in chromosomes 13p, 12, 11p, and 17p in which hypomethylation was also present. These aberrations were significant in CLL patients as compared to normal controls. Upregulation of telomerase/telomere-associated proteins was also observed in patients at early disease onset [96]. As in the case of most leukemia, *TERT* activation occurs via STAT5 mediated binding and its phosphorylation state [89,97,98]. This mechanism, although not studied extensively in CLL, may be a contributing mechanism as to how CLL progress in addition to the upregulated telomerase/telomere-associated proteins and telomeric length dysregulation.

Furthermore, in terms of DNA methylation status it was found that methylation of *TERT* promoter was crucial in different stages of leukemia development as it confers different functional advantages. As mentioned earlier, telomeric shortening is a contributing factor in disease onset. It was found that this is crucial in telomerase overexpression as promoter methylation contributes to increase in initial telomeric activity in order to repair shortened telomerase during disease onset, a mechanism of immortalization in cancers [99]. This asserts the importance of *TERT* studies in CLL and its contributing mechanism via promoter methylation, which would allow us to better understand and properly treat this malignancy via direct *TERT* targeting. It is interesting to note that *TERT* dysregulation leads to disease development as previous studies in other types of leukemia associates this with chemo-resistance, disease progression, and poor prognosis [100,101].

Dysregulation of *TERT* in CLL was also observed via single nucleotide polymorphisms (SNPs). Different SNPS led to different potential in *TERT* induced self-renewal [102]. It was reported that SNPs rs2736100, rs2853690, rs33954691, and rs35033501 resulted in different outcomes in affected patients. SNP rs35033501 was found to be most prominent in patients bearing CLL and was associated with longer telomeres. This may implicate *TERT* hyperactivity in these SNPs bearing patients in which may contribute to disease development. The same report also mentioned that the rs2736100 SNP were associated with early stage of disease (I-II), while rs35033501 was associated with late stages. Whether this mutation is acquired following disease progression was not mentioned, however it is interesting to note that different SNPs result in different prognosis in patients [103].

Additionally, variation within the *TERT* and *TERT C* gene also contributed to an increase in telomeric ends. It was found that *TERTC* rs10936599 SNP with C allele had a significant association with CLL development as high allele frequency was observed in CLL patients. The same study also observed that *TERT* rs2736100 occurring in patients bearing CLL was associated with increased CLL risk [104]. How this contributes to leukemogenesis is still understudied but we could link this to an increase in TERT activity as observed in colorectal cancer [105].

**Figure 3:**
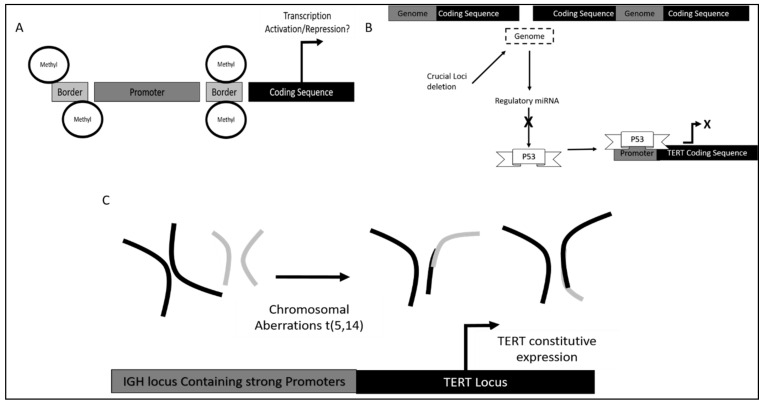
Control of TERT activity and expression occurring in CLL. (**A**) Promoter methylation status; (**B**) chromosomal deletion containing loci encoding for transcription factor suppressors; (**C**) chromosomal aberrations resulting in fusion of critical cis-regulatory elements with *TERT* coding sequence.

Chromosomal aberrations were also found to contribute to *TERT* dysregulation in CLL. This includes the *TERT/CLPTM1L* locus in chromosome 5p15 and t(5,14) translocation, which resulted in the fusion of *TERT* to the *IGH* gene [106]. The *IGH* locus, which contains strong promoters, may have enhanced *TERT* activation as a significant increase in *TERT* expression was observed in CLL harboring this translocation [107]. Besides that, it was also found that deletion on chromosome 13 at locus q14 (13q14del) also contributed to better prognosis in patients. This locus contains the coding sequence for miR-15a/16-1 cluster which target *TERT* suppressor p53 [108,109]. The mechanism is illustrated in Figure 3.

In the case of drug studies against *TERT* in CLL it was found that targeting factors crucial in telomerase assembly may contribute to increase in sensitivity of CLL treatment with fludarabine. A study showed that using a telomerase inhibitor Imelstate (IS) with fludarabine does not lead to reduction in telomerase activity. However, another report on the association between NOP17 and telomerase assembly suggested that IS and fludarabine treatment dysregulate NOP17, resulting in impairment of TERT assembly at telomeric ends [110]. Furthermore, IS was also found to suppress telomerase activity, suggesting potential of IS treatment of CLL via synergy between chemo-drug and the compound [111]. Though studies on drug directly targeting TERT are not extensive, we believe that co-treatment may result in better efficacy of chemotherapy. Co-treatment may be done via directly targeting TERT as mentioned earlier in CML or by targeting indirect factors crucial of telomeric extension. For example, BRD4 inhibitor could significantly reduce telomeric ends length which may contribute to cellular senescence [112]. Using this drug alone may not significantly contribute to CLL inhibition, therefore co-treatment with chemo drug fludarabine may be applied which may result in a synergistic effect of CLL suppression as observed with IS. As the mechanisms of drug action on direct *TERT* regulation in CLL is not extensively studied, this field of study may be worth exploring in order to better understand *TERT* dysregulation in CLL.

### 5.3. TERT Dysregulation and Clinical Implications in Acute Leukemias

Acute leukemia refers to an aggressive form of leukemia that typically progresses rapidly over a short period of time. It can be further subdivided into acute myeloid (AML) and acute lymphoid leukemia (ALL) [113]. *TERT* dysregulation that has been identified as one of the contributors to the pathogenesis of leukemia and has been reported in both AML and ALL.

#### 5.3.1. *TERT* Dysregulation and Clinical Implications in Acute Myeloid Leukemia

Acute myeloid leukemia (AML) is a type of leukemia that arises from the myeloid progenitor of hematopoiesis and gives rise to immature blasts in the bone marrow and/or peripheral blood. Lin et al. (2016) reported that *TERT* overexpression could promote the development of t(8;21) AML by working in tandem with *AML-ETO* possibly through two main mechanism: telomere integrity maintenance and inhibition of replicative senescence [114]. In addition, gene amplification which is a common characteristic of cancer malignancy was also detected in AML. *TERT* gene amplification was identified through fluorescence in-situ hybridization (FISH) in AML samples and was positively correlated with higher telomerase activity, which may contribute to AML leukemogenesis [80,115,116,117]. Single nucleotide variants (SNV) have also been identified as a common finding in cancers [118]. In a study by Mosrati et al. (2015), two *TERT* SNVs of CC genotypes (rs2853669 and rs2736100) that are associated with an increased risk of AML were identified [119]. rs2736100 is located at intron 2 of the *TERT* gene, whereas rs2853669 is positioned at the *TERT* promoter. Inflammation is one of the hallmarks of cancer and it was found that CC variant of rs2853669 was linked to increased level of inflammatory cytokines IL-6 and TNF-α, which may contribute to cancer malignancy. In the past decade, many studies have sought to identify *TERT* variants in various types of cancer, as these variants could be the driver of carcinogenesis. One of the most commonly found mutation in AML is G>A conversion in codon 1062 situated at exon 15 of the *TERT* gene, resulting in alanine>threonine substitution (A1062T) [120,121,122,123]. AML patients with A1062T *TERT* mutation had a relatively poorer prognosis as this mutation is associated with shorter overall survival, higher relapse incidence, and greater rate of treatment-related toxicity [120,121]. Epigenetic regulation of *TERT* has been the focus of many studies as it is believed to aid in the malignant transformation of cells [54]. Hypermethylation of the *TERT* promoter results in TERT transcription activation that subsequently leads to higher telomerase activity [124]. According to a study by Zhao et al. (2016), *TERT* proximal promoter and a partial exon 1 (TERTpro/Ex1) region exhibits CpG site hypermethylation in AML cell lines and primary blasts [125]. This distinct methylation profile could serve as a prognostic factor in AML as it is associated with shorter overall survival and drug resistance. Apart from the above regulation, *TERT* is also regulated by oncogenic signaling FLT3-ITD (FMS-Like Tyrosine Kinase 3 Internal Tandem Duplication). FLT3, a type of receptor tyrosine kinase is often mutated in AML with internal tandem duplications within the juxtamembrane domain. Compelling evidence has implicated the role of FLT3-ITD mutation in leukemogenesis especially in the case of AML [126]. Recently, it has come to light that FLT3-ITD mutation could induce the expression of *TERT*, resulting in increased telomerase activity that contributes to AML pathogenesis [127]. Another method of telomeric elongation identified in promyelocytic leukemia (PML) (a form of AML) is alternative lengthening of telomeres (ALT) [128]. The study by Osterwald et al. (2015) elucidated that the formation of nuclear bodies at telomeres leading to ALT were a result of concerted effects by 29 proteins involved in DNA repair, protein sumoylation, and organization of chromatins and telomeres. Changes mediated by these nuclear bodies include the increase in phosphorylated ataxia telangiectasia mutated kinase and stimulation of DNA damage responses, which promote maintenance of telomeres. Furthermore, in acute promyelocytic leukemia (APL), it was discovered that disease outcome correlated with alterations on the distal domain of the *hTERT* promoter [129]. In this study, hypomethylation and deposition of H3K4Me3 at the approximate region of 5kb upstream of the transcription start site was associated with activation of *hTERT*. These results, therefore, point to the combinatory interaction of *hTERT* promoter methylation, histone modifications, and chromatin accessibility in dysregulation of *hTERT* transcription. A study on childhood AML further delineated that there exist a correlation between telomerase activity and methylation of cyclin-dependent kinase inhibitor 2B (CDKN2B) CpG islands [130]. It was revealed that in the cohort of patients with methylated CDKN2B, their mean levels of telomerase were 49.09 ± 44.43 total product generated (TPG), as compared to 29.99 ± 32.43 TPG in patients without CDKN2B methylation. This finding suggests that hypermethylation in CDKN2B CpG islands is positively linked to the increase of telomerase expression.

Conventional chemotherapy is one of the therapeutic approaches used to treat AML patients, whereby it is common that a combination of chemotherapeutic drugs is used. Each of the chemotherapeutic drug aims to target and inhibit the cancer cells via various mechanisms. One of the mechanisms targeted by a commonly used drug for AML, 5-azacytidine (5-AZA) is DNA methylation. 5-AZA, a DNA methyltransferase inhibitor (DNMTI), has been shown to downregulate *TERT* expression and decrease telomerase activity in AML patient samples and cell lines [131]. Similarly, another study reported that DNMTI 5-Aza-2′-deoxycytidine (DAC) treatment on AML patient samples and cell lines demonstrated a reduction in *TERT* expression and telomerase activity [132]. However, the mechanism by which 5-AZA and DAC regulates *TERT* expression in AML has not been fully described to date.

In addition, small molecule inhibitors have been emerging as potential therapeutic alternatives to treat AML patients. This is exemplified by Triptonide (diterpene triepoxide), a small biological molecule derived from the herb *Tripterygium wilfordii*, that demonstrated antileukemic activity by inhibiting c-Myc transcription and its downstream target, *TERT* in AML cells [133]. It is also important to note that triptonide not only suppressed the expression of senescence-inhibiting genes but also activated the expression of senescence-promoting genes, such as *P16* (*CDKN2A*) and *P21* (*CDKN1A*). Thus, triptonide is known as “multiple-hits” senescence-promoting agent. Furthermore, a tyrosine kinase inhibitor (TKI), PKC412 exerts its inhibitory action on *TERT* via FLT3-ITD in a c-Myc dependent manner that functions as the transcription factor of *TERT* [127].

TERT-targeted immunotherapy has also become the subject of many cancer therapeutic studies. A study by Sandri et al. (2017) demonstrated that TERT865-873-specific, TCR-engineered T-cell lymphocytes were able to halt AML progression in vivo [134]. The main idea behind this therapy, known as adoptive T-cell therapy (ACT), is to increase the affinity and specificity of T-cell receptor (TCR) of T cells to tumor associated antigen (TAA). It is rather interesting to note that TERT865-873-specific, TCR-engineered T-cell lymphocytes were able to distinguish between AML blasts and peripheral blood mononuclear cells (PBMC), indicating its high cell recognition specificity. Khoury et al. (2017) found that treatment of AML patients with *hTERT* expressing autologous dendritic cells (TERT-DC) was able to improve their overall survival rate associated with favorable recurrence-free survival [135]. hTERT-DC is comprised of dendritic cells (DC) coupled with mRNA-encoding telomerase (hTERT) and the lysosomal targeting signal 38 lysosomal-associated membrane protein (LAMP). Thus, it can be seen that TERT-specific immunotherapies are an effective therapeutic approach in combating AML.

#### 5.3.2. *TERT* Dysregulation and Clinical Implications in Acute Lymphoblastic Leukemia

Acute lymphoblastic leukemia (ALL) is a type of leukemia that arises from the lymphoid progenitor of hematopoiesis and gives rise to immature blasts in the bone marrow and/or peripheral blood. Previous studies have reported that the expression of *TERT* is generally upregulated in ALL. This indicates that *TERT* overexpression could play a role in leukemogenesis [116,136]. *TERT* gene amplification was detected through FISH analysis in ALL patient samples coupled with increase in telomerase activity [116]. Single nucleotide polymorphism (SNP) is present at a frequency greater than 1% in the general population [137]. Sheng et al. (2013) discovered that in the *TERT* promoter region, SNP, rs2735940 with C to T substitution, is able to induce *TERT* expression by upregulating its transcriptional activity in ALL [138]. Not surprisingly, this specific SNP is associated with increased risk of pediatric ALL among Chinese population thus making it as a biomarker in ALL diagnosis. However, contradicting result was reported by Eskandari et al. (2018) whereby it was found that *TERT* promoter region SNP, rs2735940 with C to T substitution, does not increase the risk of ALL [139]. Furthermore, epigenetic regulation of *TERT* in pediatric ALL has been demonstrated by Borssén et al. (2011) [140]. *TERT* promoters in 24% of ALL patient samples shows greater methylation pattern as compared to normal bone marrow samples. However, findings from this study have established that there is a negative correlation between *TERT* promoter methylation status and *TERT* gene expression. It is also important to note that the incidence of methylation is higher in T cell-ALL than B-cell precursor ALL, indicating that *TERT* promoter methylation can be used to differentiate between specific ALL subtypes.

TERT-targeted therapies are quickly gaining popularity as a therapeutic option due to the role of TERT as a universal tumor associated antigen (TAA). BIBR1532, a small biological molecule specific for telomerase inhibition, has been studied by Bashash et al. (2017) for its application in pre-B ALL [141]. BIBR1532 was able to suppress *TERT* expression and telomerase activity in vitro in a dose-dependent manner. In addition, BIBR1532 suppressed the expression of c-Myc that acts as a transcriptional activator of *TERT*. Doxorubicin, a commonly used chemotherapeutic drug, was found to exert the same inhibitory action as BIBR1532 on *TERT* and *c-Myc*. This study also reported that the combination of doxorubicin together with BIBR1532 showed a greater inhibition of *TERT* and *c-Myc* transcription as compared to its individual counterparts. Thus, this combination therapy can be an effective therapeutic approach for pre-B ALL.

TERT-targeted immunotherapy is being studied for its application in ALL. According to a study by Sandri et al. (2017), TERT865-873-specific, TCR-engineered T-cell lymphocytes were able to halt ALL progression in vivo [134]. Similar to AML, hTERT865-873-specific, TCR-engineered T-cell lymphocytes were able to distinguish between ALL blasts and B cells from peripheral blood mononuclear cells (PBMC). Therefore, it can be concluded that TERT specific T cells have shown promising outcomes as next generation treatments for ALL.

## 6. Challenges and Opportunities in Targeting hTERT in Cancer Therapy

To date, a vast majority of experimental treatments against hTERT have not been able to display promising clinical benefits in studies on leukemia. This is primarily due to several unresolved issues, including the absence of relevant animal models, the inability to obtain high resolution molecular structure of the human telomerase enzyme, and the development of resistance towards inhibitor-based therapies [142].

The challenge in developing mouse models for targeting TERT mainly stems from global expression of telomerase in adult somatic cells [143]. Currently available strains exhibit far longer telomeres than observed in humans and would prove unsuitable for replicative senescence studies relating to TERT.

Elucidation of the molecular structure of telomerase has also been hampered by failure to isolate the enzyme in large quantities due to low cellular abundance, which therefore impedes downstream purification and crystallographic works [142]. This has inevitably hindered mechanistic studies and drug design efforts.

In addition, studies have revealed that drugs targeting *hTERT* require extended durations to manifest clinical effects as they rely on the attrition of telomeres to arrest the cell cycle [142]. Off target effects could also affect stem and progenitor cells which express *hTERT*. Furthermore, malignant cells may also escape treatment due to their ability to survive with low levels of *hTERT* and potential to evolve resistant clones, such as adapting telomere maintenance to the ALT pathway as earlier mentioned, before treatment becomes effective.

In order to address the aforementioned pitfalls, potential strategies have been improvised to overcome each of the shortcomings. TERT inhibitors with high potency, which are capable of fully depleting telomerase activity, have been proposed to exert greater selective pressure and overcome resistance in cancer cells [142]. This strategy may also be more effective against leukemic cells with characteristically shorter telomeres as replicative senescence could be manifested sooner. Furthermore, increasing efforts have also been made to acquire higher resolution molecular structure of TERT including a recent study employing cryo-electron microscopy which successfully identified that human telomerase is able to take on a monomeric structure when bound to its substrate [144]. In terms of preclinical studies, it has been proposed that using patient xenografts derived from early passages could work as an effective model which more closely recapitulate the genetic complexity in human cancers. This would also be able to overcome discrepancies arising from species-specific *TERT* expression [142].

## 7. Approaches Targeting Telomerase in Preclinical and Clinical Trials

### 7.1. Immunotherapies

The revelation that cancer cells express TERT epitopes as a surface marker enabled the development of immunotherapies to target telomerase [145]. The devised strategies thus far include vaccines, adoptive cell transfer, and oncolytic viruses.

TERT-based vaccines rely largely on eliciting recognition and adaptive immune responses in T-cells against telomerase epitopes via MHC class I or II molecules [142]. A phase I/IIa clinical trial in patients with metastatic hormone-naïve prostate cancer demonstrated that the UV1 TERT vaccine was able to elicit measurable immune responses in 18/21 or 85.7% of the cohort [146]. Noteworthy, in 45% of patients, MRI indicated no persisting tumor in the prostatic gland. However, in another phase III trial on advanced pancreatic cancer patients, the TERT vaccine GV1001 did not display significant survival advantage as compared to chemotherapy [147]. These trials exemplify that TERT vaccines alone may not be sufficient in controlling disease progression. More recently, a preclinical study evaluating the co-administration of TERT vaccine with immune checkpoint blockade (anti-CTLA-4) has revealed that a synergistic effect could be achieved, resulting in suppression of tumor growth accompanied by increased survival (Table 1) [148]. It was also highlighted that checkpoint blockade alone or vaccine alone were far from achieving the outcome of the combined treatment, thus rationalizing further development of this strategy.

Adoptive cell therapy was developed to use the patient’s own T lymphocytes with anti-cancer activities (either primed or engineered) in an ex vivo expanded manner. Recent studies have shown that adoptive cell transfer with high avidity telomerase-specific T-cells were able to suppress prostate cancer progression and improve survival of preclinical cancer models [149]. However, autoimmunity was present as evident by depletion of B-cell, albeit the transient observation. Further studies on AML/CML demonstrated that T-cells engineered to express receptors with high avidity to hTERT resulted in suppression of the disease in humanized mouse models [150].

Oncolytic adenoviruses such as telomelysin have been developed to infect and replicate selectively in malignant cells through the control of *hTERT* promoter driven E1 gene expression [142]. Preclinical assessments found that treatment with telomelysin repressed xenographic gastrointestinal tumor growth via induction of cancer cell death [151]. Additionally, it was also found in the same study that non-immunogenic cancer cells were sensitized to anti-PD1 immunotherapy upon treatment with telomelysin. This finding justifies further investigations into combinational therapy of oncolytic viruses with immune checkpoint blockades.

### 7.2. Oligonucleotide Inhibitors

Oligonucleotide inhibitors such as imetelstat work by complementary binding to the TERC template, thereby acting through competitive inhibition of telomerase. Its mechanism of action lead to attrition of telomeres, which prompts DNA damage response and ensuing cell death. However, hematological side effects were observed in a dose-dependent manner when imetelstat was administered into patients with solid tumors [152]. A later phase II clinical trial further found that progression-free survival (PFS) and overall survival (OS) were not improved with treatment [153]. Despite these findings, it was observed that patients with the shortest telomeres benefited from imetelstat as indicated by a trend towards improving survival. Accordingly, imetelstat has been repurposed for myeloproliferative disorders such as myelofibrosis, which are characteristically known to have shorter telomeres [111].

### 7.3. Small Molecule Inhibitors

BIBR1532 is a small molecule inhibitor that works by non-competitive binding to a conserved hydrophobic pocket (FVYL motif), which impairs interactions between TERT and TERC [154]. Treatment with BIBR1532 has produced promising preclinical results despite requiring extended treatment due to its reliance on telomere shortening to induce replicative senescence and characteristically poor pharmacokinetics. Moreover, treatment on leukemic cells lead to observation of acute cytotoxicity which was found independent of telomere shortening. Further investigations revealed the mechanism of action involved p53 dependent DNA damage response, which mediated rapid induction of telomeric dysfunction [155].

### 7.4. Indirect Inhibitors

#### 7.4.1. G-Quadruplex Stabilizers

Telomeric regions rich in guanine have been observed to form secondary structures known as G-quadruplex via Hoogsteen hydrogen bonding. G-quadruplex stabilizers are able to prevent access of DNA helicases to telomeres and therefore increase propensity towards DNA damage response and subsequent cell death [156]. One such drug tested in preclinical studies using leukemic xenograft models is telomestatin which have shown to inhibit telomerase activity, increased apoptosis of cancer cells and reduced the size of tumors [156]. However, concerns have been raised about the safety of G-quadruplex stabilizers as it has been found through computational prediction that the human genome could form more than 300,000 G-quadruplex structures at various sites including non-telomeric regions [157]. Further studies in this area should endeavor to elucidate the conformation of G-quadruplex in telomeres and design specific G-quadruplex ligands that selectively target telomeric regions [158].

#### 7.4.2. Nucleoside Analogues

Nucleoside analogues including 5-fluoro-2′-deoxyuridine thiophosphate (5-FdU) and 6-thio-2′-deoxyguanosine (6-thio-dG) function as uncapping agents which prevent recruitment and formation of the shelterin complex on telomeres [159,160]. The incorporation of these analogues into the telomeric structure induces dysfunction of the telomere ends leading to activation of the DNA damage response. Nucleoside analogues have been shown to induce minimal side effects on normal human cells whilst exerting potent cytotoxicity on xenografts of lung cancer [161], medulloblastoma [162], and melanoma [163] in preclinical models. Considering these agents function independently of telomere lengths, their use is not limited by heterogeneity of this factor found in most cancer cells [142]. Furthermore, it has been discovered that nucleoside analogues acutely induce apoptosis in transformed cells which excludes prolonged use and undesired suppression of telomerase in stem and progenitor cells. However, due to its rapid response, neutropenia has been documented [159]. Further, it has been found that 6-thio-dG also causes mitochondrial antioxidant adaptive response, which is able to prevent apoptosis in melanoma cells [164]. This effect can be counteracted by including mitochondrial Hsp90 inhibitor alongside nucleoside analogue treatment to induce higher apoptotic effect in cancer cells.

## 8. Conclusions and Future Perspectives

In summary, many preclinical and clinical studies on leukemia have concluded that TERT plays a pivotal role in disease progression and deteriorating prognosis of patients. Studies aimed at determining the factors influencing *TERT* expression during leukemogenesis and the stage where this gene is upregulated has contributed immensely to the understanding of the fundamental roles of TERT in various forms of leukemias. Additionally, it has also been proven that different mechanisms are involved in the expression of this gene including transcriptional, genetic, and epigenetic regulators. These mechanisms of activation and dysregulation of *TERT* in onco-hematological diseases are progressively being elucidated and in recent years, researchers have deciphered many novel variants, modifications, pathways, and mediators leading to its overexpression. The latest findings have identified mutations in the *hTERT* gene, methylation of its promoter, and microRNAs targeting its transcript as leading areas of fundamental studies and highly promising for therapeutic development. Indeed, progress in clinical trials have determined that chemical drugs and biomolecules targeting various regulators of *TERT* have immense potential in controlling the proliferation and metastasis of leukemias, primarily via arrest of cellular division. Noteworthy, in recent years there have been renewed interests in targeting hTERT as a therapeutic strategy due to developments in several areas including immunotherapies, drug discovery, gene therapy, and radiotherapy. Combination of agents specific to TERT and other anti-cancer drugs have also been widely explored with many studies indicating synergistic effects, reduced dose requirements and decreased side effects (Table 1). The survival outcomes of leukemic patients have improved in recent years, and it is expected that therapies targeting TERT and their combinations with other anti-cancer agents could further improve the prognosis of patients especially those with TERT-dependent leukemias.

## Figures and Tables

**Table 1:** Combinatorial effects of co-administering TERT targeted treatment with other anti-cancer agents. Potential benefits of the combined therapies indicate synergistic effects leading to tumor regression via multiple pathways.

No	Type of TERT Treatment	Combination Therapy	Potential Benefits	Reference
1	*hTERT* DNA vaccines	Immune checkpoint blockade (CTLA-4 and PD-1)	Immune checkpoint inhibitor potentially modifies the immune regulatory environment of tumors (including expansion of T cells) to synergize with TERT DNA vaccines, leading to synergistic anti-cancer activity	Duperret et al., 2018 [148]
2	*hTERT* epitope peptide vaccine	Metronomic chemotherapy	Combinatorial approach increases specific T cell response and decreased Treg frequency	Tagliamonte et al., 2015 [165]
3	Oncolytic adenovirus (Telomelysin)	Immune checkpoint blockade (anti-PD-1/anti-PD-L1)	Prior treatment with telomelysin sensitizes cancer cells to subsequent immune checkpoint inhibitors administration, which synergises increased tumor regression	Kanaya et al., 2018 [151]
4	Retroviral-delivered hTERT-specific siRNA	Chemotherapy (topoisomerase inhibitors or bleomycin) and ionizing radiation (IR)	siRNA treatment induced replicative senescence, decreased telomerase activity, attenuated tumorigenicity, and sensitized cancer cells to chemotherapeutic agents and IR	Nakamura et al., 2005 [166]
5	Lentiviral-delivered hTERT-specific shRNA	Doxorubicin (topo isomerase 2 inhibitor)	hTERT shRNA sensitized cancer cells to doxorubicin through decreased proliferation rates and autophagy	Romaniuk-Drapała et al., 2021 [167]
6	Nucleoside analogue (6-thio-dG)	Mitochondrial Hsp90 inhibitor (Gamitrinib)	Nucleoside analogue treatment with 6-thio-dG triggers a mitochondrial antioxidant adaptive response, which can be counteracted with mitochondrial Hsp90 inhibitor for higher apoptotic effect in cancer cells	Reyes-Uribe et al., 2018 [164]
7	Telomere Homolog Oligonucleotide (T-Oligos)	Chemotherapy (Vincristine, Cyclophosphamide, Adriamycin, Prednisone)	Reduced combinational doses of T-oligos and chemotherapy stimulate cell cycle arrest, senescence, and apoptosis via caspase-3 and p53 activation.	Longe et al., 2009 [168]
8	Small molecule *hTERT* inhibitor (BIBR1532)	Imatinib mesylate (IM) (tyrosine kinase inhibitor)	IM has been determined as an effective drug against CML but potentiates leukemogenesis via STAT5 phosphorylation leading to *TERT* activation. Co-administration of TERT inhibitor such as BIBR1532 could ameliorate this side effect.	Celeghin et al., 2016 & Yamada et al., 2011 [91,94]
9	Small molecule *hTERT* inhibitor (BIBR1532)	Doxorubicin (topo isomerase 2 inhibitor)	Induction of synergistic anti-cancer effect via p73 activation, repression of proliferation by p21-mediated G1 arrest, downregulation of *c-Myc*, *TERT* and *survivin*, increase ROS leading to caspase-dependent apoptosis, and enhancing pro-oxidant effect of doxorubicin.	Bashash et al., 2017 [141]
10	Small molecule *hTERT* inhibitor (BIBR1532)	Ionizing radiation (IR)	BIBR1532 increased radiosensitivity of cancers by enhancing IR-mediated mitotic catastrophe, senescence, and apoptosis via induction of telomere dysfunction and inhibit the *ATM/CHK1* pathway	Ding et al., 2019 [169]

## Data Availability

Not applicable.
